# Clinical Holistic Medicine: The Existential Crisis—Life Crisis, Stress, and Burnout

**DOI:** 10.1100/tsw.2005.40

**Published:** 2005-04-06

**Authors:** Søren Ventegodt, Isack Kandel, Shimshon Neikrug, Joav Merrick

**Affiliations:** ^1^ Nordic School of Holistic Health and Quality of Life Research Center, Teglgårdstræde 4-8, DK-1452 Copenhagen K, Denmark; ^2^ Faculty of Social Science, Academic College of Judea and Samaria, Ariel, Israel; ^3^ National Institute of Child Health and Human Development, Faculty of Health Sciences, Ben Gurion University of the Negev, Beer-Sheva, Office of the Medical Director, Division for Mental Retardation, Ministry of Social Affairs, Jerusalem, Israel

**Keywords:** quality of life, QOL, philosophy, human development, holistic medicine, public health, holistic health, holistic process theory, life mission theory, group therapy, Denmark

## Abstract

The triple and parallel loss of quality of life, health, and ability without an organic reason is what we normally recognize as a life crisis, stress, or a burnout. Not being in control is often a terrible and unexpected experience. Failure on the large existential scale is not a part of our expectations, but most people will experience it. The key to getting well again is to get resources and help, which most people experience with shame and guilt. Stress and burnout might seem to be temporary problems that are easily handled, but often the problems stay. It is very important for the physician to identify this pattern and help the patient to realize the difficulties and seriousness of the situation, thus helping the patient to assume responsibility and prevent existential disaster, suicide, or severe depression. As soon as the patient is an ally in fighting the dark side of life and works with him/herself, the first step has been reached. Existential pain is really a message to us indicating that we are about to grow and heal. In our view, existential problems are gifts that are painful to receive, but wise to accept. Existential problems require skill on the part of the holistic physician or therapist in order to help people return to life—to their self-esteem, self-confidence, and trust in others. In this paper, we describe how we have met the patients soul to soul and guided them through the old pains and losses in order to get back on the track to life.

## INTRODUCTION

Events that overwhelm us emotionally and "shock" us may have very serious consequences for our lives. Typical examples are painful, violent, and sexual assaults; incest; war traumas; accidents with near-death experiences; and other serious events. Actually, seemingly harmless events (such as a stressful exam, scolding, rejection, or neglect in childhood) or even terrible things that we witness, but do not experience for ourselves, can move us and overwhelm us enough to make the "shock" reside in us and make us think, feel, and act in irrational and disturbed ways. Many small events where we let go of our sound and true self little by little (for example, in the workplace) have similar undermining effects on our life. The essence of what we usually give the scientifically vague terms "life crisis", "spiritual crisis", "stress", and "burnout" are basically the triple loss of quality of life, health, and ability to function.

The holistic process theory[1] claims that the physical, emotional, and mental aspects of the trauma can be handled in a simple way, by helping the patient to feel, understand, and let go of negative beliefs and decisions. The acute trauma is the easiest to treat, as the patient will often heal spontaneously with a little support and holding. Old traumas, such as multiple rape in childhood, are often so repressed that they will not resurface without massive "holding" and support of the patient with a particularly close and trusting relationship from a loving and caring physician or other therapist. Love, trust, and holding are central concepts in our style of holistic medicine[2,3,4,5,6,7,8,9,10,11,12,13,14,15,16,17,18,19,20,21, 22,23,24] and discussed in a number of our books and papers[25,26,27,28,29,30,31,32,33,34,35,36, 37,38,39,40,41,42,43,44]. Holistic medicine is basically about providing sufficient support in the therapeutic situation to enable the patient to confront the old painful traumas in his or her life. The holding takes five forms: awareness to the mind, respect to the feelings, care to the body, acknowledgment to the wholeness of the person (the "soul"), and acceptance of the physical aspect, the gender, and the sexuality of the person.

The events of life seem to be controlled by a strange inner logic. Our consciousness seems to be tied up in certain patterns manifested as problematic themes of life with which we struggle. These themes appear as painful events with mysterious problems and these events seem to repeat themselves indefinitely until the moment that we fully understand our own part in them. We all know the cases similar to those of women who keep falling for men who eventually beat them. Not until she realizes her part in the pattern can she escape from it. Fortunately, it is not that difficult to sort out such themes in life, although it is very painful to acknowledge one's own responsibility for them. When the inescapable feeling of guilt finally wears off, we often also get rid of a number of emotional disturbances. The tendency towards depression, for example, may cease.

People react very differently to traumatic events. Some women recover from rape in a relatively short time, while others fall apart and are only able to get themselves together again after several years of therapy. Some victims of violence return from a night out with a broken nose and laugh about it, apparently suffering no harm from the incident, while others become frightened of other people and public places and need long-term treatment to recover. Basically, you can say that everybody has an inner physician or healer, but not everybody knows how to use "him". When the self-healing process is stuck, then the holistic physician comes into play.

## CLINICAL HOLISTIC MEDICINE

The life mission theory[25,26,27,28,29,30] states that everybody has a purpose of life or huge talent. Happiness comes from living this purpose and succeeding in expressing the core talent in your life. To do this, it is important to develop as a person into what is known as the natural condition, a condition where the person knows himself and uses all his efforts to achieve what is most important for him. The holistic process theory of healing[1,45,46,47] and the related quality of life theories[39,40,41] state that the return to the natural state of being is possible whenever the person gets the resources needed for existential healing. The resources needed are "holding" in the dimensions of awareness, respect, care, acknowledgment, and acceptance with support and processing in the dimensions of feeling, understanding, and letting go of negative attitudes and beliefs. The preconditions for holistic healing to take place are trust and the intention for the healing to take place. Existential healing is not a local healing of any tissue, but a healing of the wholeness of the person, making him much more resourceful, loving, and knowledgeable of his own needs and wishes. In letting go of negative attitudes and beliefs, the person returns to a more responsible existential position and an improved quality of life.

## EXISTENTIAL PROBLEMS ARE GIFTS

Generally, personal problems are difficulties in achieving what we want in life. Because deep inside us there is something that we desperately want — the purpose of life — we often seem to face great difficulties. Painful experiences can easily make us change course. We lie about what we want and, in so doing, fail our purpose in life.

When life is painful, it is very tempting to disclaim responsibility and escape. We may run away from the pain, but cannot remove it and we carry it around deep inside us. Then, as we try to return to our original, true course, the same difficulties show up again. The old pains and life lies resurface from the subconscious mind and distort our entire life. Indeed, the subconscious implies that we are not aware of what lies buried there because of repression and denial. The only trace of what we once suffered is the pain, suffering, anxiety, or however way the disturbance is manifested.

From this point of view, existential pain is a message to us indicating that we are about to be healed. We are about to pick up something precious from which we once fled. We are about to rediscover something that we still need, now more than ever. In our view, existential problems are gifts. They are gifts that are painful to receive, but wise to accept. It requires skill on the part of the holistic physician or therapist to help people return to life — to their self-esteem, self-confidence, and to trust in others. We need to meet the patient or client soul to soul, take him by the hand, and guide him through the old pain or loss and back to life. That is the essence of holistic medicine.

Improving one's life perspective is connected with having to let go of old perceptions to which one has become attached. It is connected with anxiety. As one lets go of one's fundamental perceptions (known as ego death), one is reborn as a true and more genuine person. The way out is the way through it. Mostly, meeting the pain face to face hurts much less than one would expect, having avoided it so long. Sometimes it does not hurt at all; rather it feels good to be relieved of the existential pain and invariably it teaches us something important about life. Once the patient's pain has shifted into acknowledgment and the negative decisions have been released, the patient has grown as a person. Sometimes a small and seemingly insignificant event may tip the balance, even though previous violent events apparently did not have any major effect.

> **Male, aged 31 years, with traumas:** Presents with palpitation and fear of death. Auscultation of the heart: normal. Slightly increased pulse, which soon decreases during the consultation. One month ago the patient nearly died together with his whole family in a car accident, in which the car overturned. None of the family members were injured, but the 7-year-old son is still very frightened. We talk about the fact that we are mortal and that life is precious and fragile. Went through the incident a couple of times. The patient should write it down.

This patient moves back and forth on the timeline harmoniously and spontaneously, guided by inner logic and necessity so stereotyped commands are not required. The patient's resources are so large that he may even finish the work at home. It takes great skill to know whether the patient needs to be held by the hand or whether he can manage on his own. It is not good for the patient if the physician misjudges him and sends him home with a task that he cannot tackle alone.

## THE EXISTENTIAL CRISES IN THE HOLISTIC CLINIC — LIFE CRISES, STRESS, AND BURNOUT

Existential pain is one of our basic conditions as human beings — life hurts. Many people are unaware of the pain residing in their inner layer. They lead superficial lives that appear to be successful, although they are suffering deep inside their souls. The reason for this division of the individual into superficial layers and deep existential layers lies at the very root of our conscious nature. We can choose and when we choose, we define our own reality. We can choose to live in several worlds at the same time and we can choose to be aware of and attentive to one of these worlds and unaware of and inattentive to another world.

To a great extent, we get away with such divisions as mind and soul or feeling and reason. We function, live our lives, and things turn out the way we want them to, but not always. The existential crisis occurs if we do not reclaim resources and qualities that we have placed in the repressed layers. If we fail, we have to acknowledge that life does not provide us with what we need and want deep down. In the course of weeks, months, or years, a person who experiences an existential crisis may change from being a jolly, extroverted person who functions well into being gloomy, introverted, and socially isolated. It is as though a bomb has gone off in that person's life. Everything is turned upside down, the most fundamental things in life no longer function. This calls for a radical *clearing out*, a new orientation in life. Frequently, the person has failed in his present version and needs to renew himself

> **Male, aged 45 years, with a life crisis:** Eats very little, has no appetite. Very tired, but is constantly active. Is no longer happy. On examination: Fast-paced, very tense in all major muscle groups – neck, back, arms, legs. I (SV) see a marathon runner, running away from his own feelings. EXERCISE: Take three days off and come to a complete stop. Just be, and feel all the emotions that emerge inside. That is the problem, in my opinion.

The picture is clear — a marathon runner, running away from himself. We frequently obtain very distinct and clear pictures that, for a brief moment, reveal something essential about the patient's current problem. When the patient confronts and acknowledges the current problem, it often diminishes. Often the patient needs some time and peace to chew on the underlying gestalts. We do not hesitate to give patients a sick leave certificate for a few days to allow them to work it out. We do not care for long-term sick leave, however. In our opinion, being unoccupied for a long time does not provide any therapeutic benefit for the patient, unless the patient has a large, relevant project, like writing a complete autobiography. In that case, this may occupy the patient for 3–6 months.

> **Female, aged 26 years, with stress:** Presents with "stress" – manifesting as a "clamp" around her head – has been off work from her job as a middle manager in a large enterprise. Married to a man from the same place, talks and thinks a lot about her job in her spare time, cannot let go of it. Three EXERCISES: Let go of the job after working hours (let go physically of the uniform). Write down all your thoughts about the job on paper and leave them there until you return to work. Make an agreement with your husband not to talk about work in your spare time. Feels an extra heart beat. ECG examination normal. BP 120/85. Next appointment in one week.

Many patients claim to suffer from "stress", but we do not buy that. In our opinion, stress comprises two elements (this is the excellent stress model from the Karolinska Hospital in Stockholm): discomfort and strain. People complain about the strain, but the discomfort is the problem. There is nothing wrong with being busy, active, committed, and strained — that is human nature — but the discomfort should not be there. It occurs when we act contrary to ourselves. In this case, working at the same place as her husband is not a good idea; it means that work is the sole content of both her life and her marital relationship. That is all wrong. The term "stress" covers something completely different. Stress cannot be treated, but should be perceived as a manifestation of some kind of acute crisis. The patient has to understand herself better and sort out her life.

## LIFE CRISES

Apparently, external factors may trigger a life crisis. Sometimes, the problems accumulate in relation to work, the family with children and partner, friends and acquaintances, or the financial situation. Suddenly, immense problems may exist everywhere. Sometimes, we may even experience misfortunes from which we cannot recover completely, like being left by our partner, the death of a child, our home being gutted by fire, or a serious physical or mental illness, which makes us question our entire existence and the meaning of life. Often, the life crisis is not caused by external factors or illness, but rather inner, existential factors. The life crisis is manifested as strange and incomprehensible mental and physical symptoms: the feeling of having wasted one’s life, of not being able to realize our great dreams in life. In short, the feeling of being a failure.

Some patients suddenly feel terribly old, tired, and wonder why life has suddenly turned so gray, dull, and monotonous. Something is wrong, but they do not know what it is. Colors are no longer bright. The enjoyment of life has disappeared. Nevertheless, the patient is not just depressed. Something has gone completely wrong; something worse than transient depression. Something in life has to be changed completely. When the existential pain breaks through to the surface of the conscious mind, it is unhealthy to ignore it or suppress it from a holistic perspective. The rational approach is to take it seriously, to address it in an analytical and exploring manner and to do something about the situation. A patient experiencing a life crisis often needs help to move on. The patient needs support to summon the courage to face himself deep down in his soul. Perhaps our patient needs the physician to act as a “mirror” that will reflect him and make him see himself with greater clarity.

> **Female, aged 51 years, with problems in life:** Quality-of-life (QOL) conversation: She attended the course “Life Philosophy that Heals”. Has major problems in life – she wants to change now. List of problems: Grinds her teeth at night – the dentist is willing to arrange an occlusal splint, but the treatment is expensive. Major marital problems with her husband for six years. No sexual relations for the past year. She does not want any closeness or intimacy with him. Physical/mental health: troubled by headache, depressed for many years, never treated medically, had her uterus removed when she was 36 years old. Low back pain occasionally. Impaired memory. Describes herself as: too nice, forbidding, prim, inhibited. “I cannot express myself,” “I’m in charge.” Social, in general: Problems with her daughter-in-law who is an incest victim. In my opinion, she lacks clarity – I get the impression of a 1,000 kg rope tied into a huge knot. EXERCISE: Write your autobiography – start from the present – what happened, how did you feel, what did you decide (write down your negative decisions on paper and bring them here – let go immediately, if possible). PLAN: Gestalt therapy – 20 hours. Can be supplemented with Rosen therapy. Session with me (SV) every month or every other month.

The fact that I get the impression of a 1,000-kg rope tied into a huge knot probably means years of work for the patient and 20 h of gestalt therapy is a good start. Unfortunately, after referring the patient for gestalt therapy, we lost contact with her so we do not know how the story ends.

The following case record describes a patient who relived a trauma that made him leap half a meter out of his stomach, mentally speaking. This is very odd and difficult to understand, but apparently in the conscious mind we can adopt any vantage point. Incredible precision and intelligence in life means that distances, times — indeed any measurable qualities — are handled elegantly and smoothly, including when it comes to early traumas. Scientifically, this is disconcerting because no developed nervous system so far can be behind this, so which part of life provides the intelligence? Conventional, medical science would reject the reports as unreliable interpretations by the adult, but we sense that it is wise to believe that life does actually possess such embedded intelligence. We believe that it exists at the cellular level, but proving it is extremely difficult.

> **Male, aged 33 years, “I am nothing”:** Patient has felt completely stuck and depressed for six weeks. “I run away,” he says. We agree that a more accurate description is that he is paralysed with fear. He wants to go into process NOW. On the couch, we work with “rebirthing” [the patient breathes forcefully, heavily and regularly until the gestalt crystallises]. “There is nothing”, “I am outside”, “It’s all black” he says. And later “I am blissful out there” (“I am nothing”). Re-experience himself as a 5-month-old foetus with umbilical cord, where he is half a metre outside the stomach – there is emptiness and freedom. He lets go of the statements by means of a roll and sense that he is back in the stomach. He breathes again, and this time he breathes heavily and naturally, with his entire body. He then experiences being a rejected little boy 2 or 3 years old. EXERCISE: Write down your life’s story starting with the statements that you let go – how you were always rejected and excluded.

Life crises may sometimes refer directly back to a large, early gestalt, which can be released on the couch, for example, by means of breathing techniques as in this case. In the same way that timeline therapy is rarely necessary, breathing techniques are only necessary when we seek to overcome mental resistance here and now. This patient had been making tentative efforts for a long time and really needed a breakthrough, which he achieved.

## SPIRITUAL CRISES

A special form of life crises is the spiritual crises. They are troublesome elements in the life of the conscious individual, but such crises make sense. We are not quite true to ourselves. We do not lead our lives in accordance with our personal project of life; therefore, problems arise. The patient may be an academic or a businessman experiencing good personal growth. He may be successful professionally and personally and have many challenges at work and in life. Then, one day, everything comes to a halt. The patient has hit a wall and is unable to move on. For example, we had a patient who was an artist and who had enjoyed rapid growth for a couple of years. Then, all of a sudden, a terrible pain caught up with her that made her want to get away from it all. She never wanted to touch a paintbrush again. She needed a fundamental shift in perspective.

> **Male, aged 45 years, with spiritual crisis:** A man who had experimented with sexual tantra techniques [meditation with channelling of sexual energies] and experienced supreme ecstasy – he had made love to his goddess in the form of his partner – experienced the most devastating, unrequited love for her; total dependence, laying his emotional life in ruins. When his girlfriend, who has now left him, was having sex with another man, he is still so connected to her that he can feel everything that is going on and he suffers terribly. The crisis lasted for several years, during which he meditated on the problem until it finally wears off and he met another woman.

Religious people may experience a struggle within them between God and the Devil, eventually making them wonder whether they are going insane. Such patients usually experience peace following a nighttime visit by the Holy Spirit. Patients with known mental disorders fall into what we would call a genuine spiritual crisis when the evil inside them appears to have won the first round and they refuse to surrender because they know that basically they are not evil. This type of crisis is extremely unpleasant for patients, but can often have a positive outcome when good ultimately triumphs. We believe that the patient should go through such crises with a maximum of personal support and care with a minimum of medication.

Spiritual crises may also be insidious; perhaps the patient shows no signs of a mental disorder or external problems. In spite of this, the patient describes a profound sense of meaninglessness or perhaps even a death wish, which has broken through from the subconscious mind and now demanding the patient's unremitting attention. The way we see it, this is about spiritual growth, about a side of the patient that has been hurt and has been hidden for an entire life and suddenly demands to be healed.

## CREATIVITY AND IMAGINATION IN THERAPY

It often requires a vivid imagination to help people in spiritual and existential crisis. There is no conventional treatment and the symptoms displayed by the patients are peculiar, to put it mildly. Such was the case with the following patient, who was so devoid of intelligence and so stagnant, that it was totally unbearable for her and for the people around her.

A young, rather overweight, and single woman with a young child came to the clinic in Copenhagen (which attracts people with all sorts of strange problems) to be cured of being a "stupid cow". She was very kind hearted and her entire life was concerned with being a good mother. For a mother that is commendable, of course, but her own life and personal development had come to a complete standstill and now she felt that all that was left of her was this stupid cow grazing in the field, while her child was sucking power and sustenance from her. In a few years, when the child would be grown up, she would still be that stupid cow, but without the calf that put at least a minimum of meaning into her life here and now. That thought tormented her terribly. She wanted to move on with her life, but felt hopelessly stuck. First, we (SV) tried to talk to her about the great philosophical questions such as the meaning of life and gratification of needs. This is what was written after one session:

> … evoked little response from her. Discussing Kierkegaard with cows simply cannot be done. I do not consider women to be stupid cows, on the contrary, a healthy woman is highly intelligent and alert. So, there was something completely wrong with her. In short, her brain was completely blocked. She had fallen into a deep slumber. In my view, she identified herself with the cow character and used up so much energy on playing the part that it took up all her mental capacity. The situation was desperate, and I struggled with it for some time. Then I had a bright idea. In principle, there are two ways of escaping from such a trap. Being caught in an existential crisis is more or less like being locked up in a cage or in prison. One has to break down the wall to become free. Sometimes, this can be done provided the person has masses of self-confidence, free energy and intellectual resources. But if it cannot be done, which was the case for this plump, young woman – who, in a way, resembled a patient with terminal cancer – an alternative solution must be sought: transcendence.
>
> If we totally accept being what we are and the way we are, where we are, we achieve taking responsibility for it. Suddenly, we become who we are, of our own free will, by our own choice – and then we are also free to choose being something else. After all, the cage in which we are trapped is our own minds, rather than external circumstances, as we tend to believe. The woman felt enslaved by her situation in life, but actually she was enslaved only by her own previous choices and decisions. She had worked herself into a corner from which she could not escape on her own. She had fallen into a "cow trap".
>
> My insight immediately led to the solution: She had to be a cow – in every sense! "Do you feel like trying an experiment, something I have not tried before, but which may help even if it seems crazy?" I asked her. Naturally, the obedient patient agreed with her doctor's advice, so I continued: "Get down on the floor with me, then we will crawl about on all fours and pretend to be cows!" Following the predictable protests and bursts of laughter, I convinced the patient to get down on the floor and for a full ten minutes we crawled about like cows, while I mooed and the patient did the same, cringing with embarrassment and laughing hysterically. "If you won't say 'moo', it will be your homework," I threatened her, and so managed to get a proper 'moo!' out of the patient. "Now, don't file a complaint about the treatment," I told her after having recovered my breath. "If you do, I'll spend two years in prison for professional misconduct as a doctor." I spoke to her two weeks later, when she was making good progress. She had crawled about on all fours, mooing, together with her child, who had had a marvellous time, until finally she realised that the reason why she was such a stupid cow was that she had once made the intellectually restrictive decision that she simply was stupid. Now, she was in the process of letting go of the decision and was already experiencing a great improvement. She laughed, when I asked her permission to publish this brief account of her miraculous recovery through "cow therapy" and she promptly gave her permission. This is the case record:

**Female, aged 32 years, with a life crisis:** First quality-of-life (QOL) session: Men make her sick. Literally had to get out and throw up on the way over here. The Rosen practitioner found: Throbbing aorta deep in the abdomen above the sacral promontory; does not feel dilated, but tender. A scan should be considered if tenderness persists. Patient has major problems with her stomach, which is too fat, too tender and nauseous. During Rosen session with the practitioner she felt immense anger, as though she were about to explode. On the couch, we work on the feelings of anger, disgust and grief, where the patient says that her father did her wrong, when she was five years old. Misses someone to care for her; we talk about how such a relationship is formed. Continue Rosen treatments.

Second QOL session: The patient wants to break away from correctness, but that is difficult as she has a young child, who needs her. The child has been very ill and she has been the good mother, which has benefited the child. She feels an urge to scream, but cannot. We practise screaming, and the patient screams with great difficulty. The diminutive scream "h" gradually becomes "ah", and ultimately a fully developed scream. The patient screams herself hoarse across the fields. The patient is stuck in a "stupid cow" gestalt, as she is present in her stomach and heart, but not in her head. She does not want to do anything, she does nothing, has no ambition or drive. The image of her is a cow at pasture. Until acceptance transcends, we practise being cows, mooing on the floor. EXERCISE: Walk about on all fours for ten minutes and "act like the cow that you are", until you can handle being what you are. On examination: The previous abdominal complaints with intense tenderness around the aorta have decreased. On palpation today there are no problems. The patient continues to see the Rosen practitioner, which is beneficial for her. She is showing good progress. She had crawled about on all fours, mooing, together with her child, who had had a marvellous time, until finally she realised that the reason why she was such a stupid cow was that she had once made the intellectually restrictive decision that she simply was stupid. Now, she was in the process of letting go of the decision and already experiencing a great improvement.

Third QOL session: Has turned her back on correctness following cow exercises. Now she does all kinds of crazy things. Her child is very happy. Went outdoors to scream and shout. It was very good and powerful. She does not believe that she has any opinion, in fact she believes that she is stupid. We talk about this and find out that she has nothing but opinions, which she needs to let go in order to reach the point, where she sees the world as it is: perfect, her natural condition. Has a large hump, excessive flexion of the thoracic spine from T1 to T8, locked into a hunchback. The "hump" hurts, she sits and stands with stooped shoulders, looking very stupid. On the couch, the hump is manipulated and straightened out with five loud snaps, and the pain in the hump disappears. In front of a mirror we work on the patient's posture, sagging breasts being transformed into nice breasts, which the patient is told to be proud of and show off next summer. Posture with retroflexion of the pelvis is straightened. With these exercises, the patient is transformed from a deformed person into a handsome woman. EXERCISE: Be proud of yourself and show it. She had breast-lifting surgery for sagging breasts when she was 20 years old. The problems with which the patient presented have been resolved, but she will not stop. Must find new goals, can return.

## BURNOUT

Why is it that a job or activity that used to be immensely interesting years ago can end up being a completely uninteresting duty that torments you day after day? Where did the interest go? What happened to that tickling sensation, the sense that it was *so* exciting and *so* challenging? What became of the enthusiastic and committed young man or woman, reading, studying, writing, and engaging in all kinds of discussions? What became of the power of concentration, the wide fingertip knowledge, creativity, good perspectives, and visions? What became of the quiet satisfaction of a job well done that lasted a surprisingly long time?

Burnout is a problem that torments hundreds of thousands of people. It is a mysterious ailment, the greatest problem being that nobody is willing to acknowledge it. The burned-out person lies to himself about how well he is doing, while knowing instinctively that that is not the case, but the fear of losing his job, status, identity, and income makes it difficult to face. Burnout is basically a consequence of a loss of interest in what one is doing due to a lack of intellectual stimulation, growth, and development. When we fail to renew ourselves, we gradually decline. Plenty of qualities and talents in a person can be developed. There is enough to learn and take an interest in, but personal development does not occur automatically. It is a laborious and often painful process. The burned-out person has to understand himself better and learn how to apply himself and his talents in a more lively and creative way. The key is a new perspective; not just in respect of working life, but of life itself. An attentive physician can help his patient recognize the true nature of the problem and how to become committed again, perhaps to something entirely different.

The individual's fundamental life philosophy is crucial for the ability to create satisfaction and development in his job. The burned-out person has to rescue his working life by incorporating the ideas of personal development in his perception of life. The patient holds great potential, regardless of the apparent degree of burnout. Contrary to what one might think, when looking into those dull eyes, it will often be possible for the patient once again to become lively and interested and have a good, long, and active working life if supported by a sympathetic person. Frequently, the burned-out patient needs extensive rehabilitation. It is important that the physician does not underestimate the extent of the problem and give the patient false hope.

**Female, aged 42 years, with burnout:** QOL session: Receptionist, aged 42 years, burnout. Feels like a 90-year-old. Looks like a 55-year-old, listless and tired. Gives a very confusing and mixed-up account of her problems, which she cannot identify herself. Worried, afraid of hurting others. Has not had sex for years, does not feel the need, she believes. Scared of contact. She looks delightful and lovely, but has made herself unnatural and messy – as though she is wearing 1,000 cactus prickles to avoid being eaten. Asks for help in becoming herself again. We talk about what it would take for her to become alive again – she has nearly made herself dead, like driving with one foot on the brake. EXERCISE: What is the meaning of life? EXERCISE: Feel your inner emotions without changing anything. Next appointment in three weeks.

This example clearly shows that the patient has to find a new meaning of life to become 20 years younger. The project is difficult, but not impossible.

## DISCUSSION

The triple and parallel loss of quality of life, health, and ability is a terrible experience. The feeling that you are not in control or on top of your own life, but lying down under your own world and hardly able to move, is often felt as a surprising defeat. Failure on the large existential scale is not a part of our expectations for our life, but most people will experience it before they die nevertheless.

The key to getting well again is to get resources or help from outside and this is often connected with shame and guilt, and is hard for most people. Taking care of the patient's difficult emotions in a vulnerable existence is the essence of the treatment for major crises in life. Stress and burnout might seem to be temporary problems that are easily handled, but all too often, the problems will not go away. They remain and after a short period of recovery, the problem returns in an even worse form. It is very important for the physician to identify this pattern and help the patient realize the difficulties and seriousness of the situations. Thus, the patient is helped to assume responsibility for his or her own future and prevents an existential disaster, suicide, or severe depression.

As soon as the patient is an ally in fighting the dark side of life[30] using the holistic approach to treatment, the physician will be able to assist in recovery. Coaching is an important concept after an acute life crisis is over. When life is painful, it is very tempting to disclaim responsibility and escape. The old pains and negative decisions lie in our subconscious mind and distort our entire lives, and as we accumulate pain and life lies throughout our lives, we gradually lose our quality of life, health, and ability[51]. Existential pain is really a message to us indicating that we are about to grow and to be healed. In our view, existential problems are gifts that are painful to receive, but wise to accept. It requires skill on the part of holistic physicians and therapists to help people return to life — to their self-esteem, self-confidence, trust in others. We have to meet the patient or client soul to soul, take him by the hand, and guide him through the old pain and loss in order for him to come back to life. That is the essence of holistic medicine.

Improving your life perspective is connected with having to let go of old perceptions to which you have become attached. Once the pain has shifted into acknowledgment and the negative decisions are released, the patient can grow as a person. Sometimes a small and seemingly insignificant event may tip the balance, even though previous violent events apparently did not have any major effect. Even the most severe life crisis can be turned into positive development if the physician understands the situation of the patient on an existential level. Using this understanding, he can support the patients in feeling, understanding, and letting go of negative convictions and beliefs[1].

## ACKNOWLEDGMENTS

This study was supported by grants from IMK Almene Fond. The quality of life research was approved by the Copenhagen Scientific Ethical Committee under number (KF)V.100.2123/91.

## References

1. Ventegodt, S., Andersen, N.J., and Merrick, J. (2003) Holistic medicine III: the holistic process theory of healing. *TheScientificWorldJOURNAL* **3,** 1138–1146.
2. Ventegodt, S. and Merrick, J. (2004) Clinical holistic medicine: applied consciousness-based medicine. *TheScientificWorldJOURNAL* **4**, 96–99.
3. Ventegodt, S., Morad, M., and Merrick, J. (2004) Clinical holistic medicine: classic art of healing or the therapeutic touch. *TheScientificWorldJOURNAL* **4,** 134–147.
4. Ventegodt, S., Morad, M., and Merrick, J. (2004) Clinical holistic medicine: the "new medicine", the multiparadigmatic physician and the medical board. *TheScientificWorldJOURNAL* **4**, 273–285.
5. Ventegodt, S., Morad, M., and Merrick, J. (2004) Clinical holistic medicine: holistic pelvic examination and holistic treatment of infertility. *TheScientificWorldJOURNAL* **4**, 148–158.

6. Ventegodt, S., Morad, M., Hyam, E., and Merrick, J. (2004) Clinical holistic medicine: use and limitations of the biomedical paradigm. *TheScientificWorldJOURNAL* **4**, 295–306.
7. Ventegodt, S., Morad, M., Kandel, I., and Merrick, J. (2004) Clinical holistic medicine: social problems disguised as illness. *TheScientificWorldJOURNAL* **4,** 286–294.
8. Ventegodt, S., Morad, M., Andersen, N.J., and Merrick, J. (2004) Clinical holistic medicine tools for a medical science based on consciousness. *TheScientificWorldJOURNAL* **4,** 347–361.
9. Ventegodt, S., Morad, M., Hyam, E., and Merrick, J. (2004) Clinical holistic medicine: when biomedicine is inadequate. *TheScientificWorldJOURNAL* **4**, 333–346.
10. Ventegodt, S., Morad, M., and Merrick, J. (2004) Clinical holistic medicine: prevention through healthy lifestyle and good quality of life. *Oral Health Prev. Dent.* **2(Suppl 1),** 239–245.
11. Ventegodt, S., Morad, M., and Merrick, J. (2004) Clinical holistic medicine: holistic treatment of children. *TheScientificWorldJOURNAL* **4**, 581–588.
12. Ventegodt, S., Morad, M., and Merrick, J. (2004) Clinical holistic medicine: problems in sex and living together. *TheScientificWorldJOURNAL* **4**, 562–570.
13. Ventegodt, S., Morad, M., Hyam, E., and Merrick, J. (2004) Clinical holistic medicine: holistic sexology and treatment of vulvodynia through existential therapy and acceptance through touch. *TheScientificWorldJOURNAL* **4**, 571–580.
14. Ventegodt, S., Morad, M., Kandel, I., and Merrick, J. (2004) Clinical holistic medicine: a psychological theory of dependency to improve quality of life. *TheScientificWorldJOURNAL* **4**, 638–648.
15. Ventegodt, S., Morad, M., Kandel, I., and Merrick, J. (2004) Clinical holistic medicine: treatment of physical health problems without a known cause, exemplified by hypertension and tinnitus. *TheScientificWorldJOURNAL* **4**, 716–724.
16. Ventegodt, S., Morad, M., and Merrick, J. (2004) Clinical holistic medicine: developing from asthma, allergy, and eczema. *TheScientificWorldJOURNAL* **4,** 936–942.
17. Ventegodt, S. and Merrick, J. (2005) Clinical holistic medicine: chronic infections and autoimmune diseases. *TheScientificWorldJOURNAL* **5,** 155–164.
18. Ventegodt, S., Flensborg-Madsen, T., Andersen, N.J., Morad, M., and Merrick, J. (2004) Clinical holistic medicine: a pilot study on HIV and quality of life and a suggested cure for HIV and AIDS. *TheScientificWorldJOURNAL* **4**, 264–272.
19. Ventegodt, S. and Merrick, J. (2004) Clinical holistic medicine: chronic pain in the locomotor system. *TheScientificWorldJOURNAL* **5,** 165–172.
20. Ventegodt, S., Gringols, M., and Merrick, J. (2005) Clinical holistic medicine: whiplash, fibromyalgia, and chronic fatigue. Submitted to *TheScientificWorldJOURNAL*
21. Ventegodt, S. and Merrick, J. (2005) Clinical holistic medicine: chronic pain in internal organs. *TheScientificWorldJOURNAL* **5,** 205-210.
22. Ventegodt, S., Gringols, M., and Merrick, J. (2005) Clinical holistic medicine: holistic rehabilitation. *TheScientificWorldJOURNAL* **5**, in press.
23. Ventegodt, S., Andersen, N.J., Neikrug, S., Kandel, I., and Merrick, J. (2005) Clinical holistic medicine: mental disorders in a holistic perspective. *TheScientificWorldJOURNAL* **5**, in press.
24. Ventegodt, S. and Merrick, J. (2005) Clinical holistic medicine: opening one’s heart in cardiovascular diseases. Submitted to *TheScientificWorldJOURNAL*.
25. Ventegodt, S., Andersen, N.J., and Merrick, J. (2003) Five theories of the human existence. *TheScientificWorldJOURNAL* **3,** 1272–1276.
26. Ventegodt, S. (2003) The life mission theory: a theory for a consciousness-based medicine. *Int. J. Adolesc. Med. Health* **15(1),** 89–91.
27. Ventegodt, S., Andersen, N.J., and Merrick, J. (2003) The life mission theory II. The structure of the life purpose and the ego. *TheScientificWorldJOURNAL* **3,** 1277–1285.
28. Ventegodt, S., Andersen, N.J., and Merrick, J. (2003) The life mission theory III. Theory of talent. *TheScientificWorldJOURNAL* **3,** 1286–1293.
29. Ventegodt, S. and Merrick, J. (2003) The life mission theory IV. A theory of child development. *TheScientificWorldJOURNAL* **3,** 1294–1301.
30. Ventegodt, S., Andersen, N.J., and Merrick, J. (2003) The life mission theory V. A theory of the anti-self (the shadow) or the evil side of man. *TheScientificWorldJOURNAL* **3,** 1302–1313.
31. Ventegodt, S., Andersen, N.J., and Merrick, J. (2003) Quality of life philosophy: when life sparkles or can we make wisdom a science? *TheScientificWorldJOURNAL* **3,** 1160–1163.
32. Ventegodt, S., Andersen, N.J., and Merrick, J. (2003) Quality of life philosophy I. Quality of life, happiness, and meaning of life. *TheScientificWorldJOURNAL* **3,** 1164–1175.
33. Ventegodt, S., Andersen, N.J., Kromann, M., and Merrick, J. (2003) Quality of life philosophy II. What is a human being? *TheScientificWorldJOURNAL* **3,** 1176–1185.
34. Ventegodt, S., Merrick, J., and Andersen, N.J. (2003) Quality of life philosophy III. Towards a new biology. *TheScientificWorldJOURNAL* **3,** 1186–1198.

35. Ventegodt, S., Andersen, N.J., and Merrick, J. (2003) Quality of life philosophy IV. The brain and consciousness. *TheScientificWorldJOURNAL* **3,** 1199–1209.
36. Ventegodt, S., Andersen, N.J., and Merrick, J. (2003) Quality of life philosophy V. Seizing the meaning of life and becoming well again. *TheScientificWorldJOURNAL* **3,** 1210–1229.
37. Ventegodt, S., Andersen, N.J., and Merrick, J. (2003) Quality of life philosophy VI. The concepts. *TheScientificWorldJOURNAL* **3,** 1230–1240.
38. Merrick, J. and Ventegodt, S. (2003) What is a good death? To use death as a mirror and find the quality in life. *BMJ.* Rapid Responses, 31 October. On-line at: http://bmj.bmjjournals.com/cgi/eletters/327/7406/66#39303
39. Ventegodt, S., Merrick, J., and Andersen, N.J. (2003) Quality of life theory I. The IQOL theory: an integrative theory of the global quality of life concept. *TheScientificWorldJOURNAL* **3,** 1030–1040.
40. Ventegodt, S., Merrick, J., and Andersen, N.J. (2003) Quality of life theory II. Quality of life as the realization of life potential: a biological theory of human being. *TheScientificWorldJOURNAL* **3,** 1041–1049.
41. Ventegodt, S., Merrick, J., and Andersen, N.J. (2003) Quality of life theory III. Maslow revisited. *TheScientificWorldJOURNAL* **3,** 1050–1057.
42. Ventegodt, S. (1995) *Quality of Life. To Seize the Meaning of Life and Become Well Again [Livskvalitet – at erobre livets mening og blive rask igen]*. Forskningscentrets Forlag, Copenhagen. [Danish]
43. Ventegodt, S. (1999) *Philosophy of Life that Heals [Livsfilosofi der helbreder]*. Forskningscentrets Forlag, Copenhagen. [Danish]
44. Ventegodt, S. (2003) *Consciousness-Based Medicine* [*Bevidsthedsmedicin – set gennem lægejournalen*]. Forskningscentrets Forlag, Copenhagen. [Danish]
45. Ventegodt, S., Andersen, N.J., and Merrick, J. (2003) Holistic medicine: scientific challenges. *TheScientificWorldJOURNAL* **3,** 1108–1116.
46. Ventegodt, S., Andersen, N.J., and Merrick, J. (2003) The square curve paradigm for research in alternative, complementary, and holistic medicine: a cost-effective, easy, and scientifically valid design for evidence-based medicine. *TheScientificWorldJOURNAL* **3**, 1117–1127.
47. Ventegodt, S., Andersen, N.J., and Merrick, J. (2003) Holistic medicine IV: principles of existential holistic group therapy and the holistic process of healing in a group setting. *TheScientificWorldJOURNAL* **3,** 1388–1400.
48. Ventegodt, S., Merrick, J., and Andersen, N.J. (2003) Quality of life as medicine: a pilot study of patients with chronic illness and pain. *TheScientificWorldJOURNAL* **3,** 520–532.
49. Ventegodt, S., Merrick, J., and Andersen, N.J. (2003) Quality of life as medicine II. A pilot study of a five-day "quality of life and health" cure for patients with alcoholism. *TheScientificWorldJOURNAL* **3,** 842–852.
50. Ventegodt, S., Clausen, B., Langhorn, M., Kromann, M., Andersen, N.J., and Merrick, J. (2004) Quality of life as medicine III. A qualitative analysis of the effect of a five-day intervention with existential holistic group therapy: a quality of life course as a modern rite of passage. *TheScientificWorldJOURNAL* **4,** 124–133.
51. Ventegodt, S., Flensborg-Madsen, T., Andersen, N.J., Nielsen, M., Mohammed, M., and Merrick, J. (2005) Global quality of life (QOL), health and ability are primarily determined by our consciousness. Research findings from Denmark 1991–2004. Accepted by *Social Indicator Research.*

## BIOSKETCHES

**Søren Ventegodt, MD,** is the director of the Quality of Life Research Center in Copenhagen, Denmark. He is also responsible for a Research Clinic for Holistic Medicine in Copenhagen and is a popular speaker throughout Scandinavia. He has published numerous scientific or popular articles and a number of books on holistic medicine, quality of life, and quality of working life. His most important scientific contributions are the comprehensive SEQOL questionnaire, the very short QoL5 questionnaire, the integrated QOL theory, the holistic process theory, the life mission theory, and the ongoing Danish Quality of Life Research Survey, 1991–94 in cooperation with the University Hospital of Copenhagen and the late professor of pediatrics, Bengt Zachau-Christiansen, MD, PhD. E-mail: ventegodt@livskvalitet.org. Website: http://www.livskvalitet.org

**Isack Kandel**, **MA, PhD,** is senior lecturer at the Faculty of Social Sciences, Department of Behavioral Sciences, the Academic College of Judea and Samaria, Ariel. During the period 1985–93, he served as the director of the Division for Mental Retardation, Ministry of Social Affairs, Jerusalem, Israel. E-mail: Kandeli@aquanet.co.il

**Shimshon Neikrug, PhD,** is a senior lecturer in Social Work at the Academic College of Judea and Samaria where he serves as the director of the Community Studies Program and teaches courses in developmental disabilities, aging, and community work. He has lectured in Social Work at Tel Aviv University and Bar-Ilan University, as well as serving as the director of the research program in the Bar-Ilan Brookdale Program. Dr. Neikrug has served as consultant to numerous programs in both the Jewish and the Palestinian sectors. He is the founder and chair of Yakir-Association for the Third Age. In that capacity, he has directed research projects on quality of life for families of children challenged by developmental disability. Dr. Neikrug has lectured at numerous professional and academic conferences in Israel, Europe, and the U.S. He is the author of a recent book, *Seniors in the Community: Knowledge and Principles* (in Hebrew), has published widely in Israeli and international journals, and has contributed chapters in edited books on disabilities and aging .E-mail nshimshon@yosh.ac.il.

**Joav Merrick, MD, DMSc,** is professor of child health and human development affiliated with the Zusman Child Development Center, Division of Pediatrics and Community Health at the Ben Gurion University, Beer-Sheva, Israel; the medical director of the Division for Mental Retardation, Ministry of Social Affairs, Jerusalem; and founder and director of the National Institute of Child Health and Human Development. He has numerous publications in the field of child health and human development, rehabilitation, intellectual disability, disability, health, welfare, abuse, advocacy, quality of life and prevention. Dr. Merrick received the Peter Sabroe Child Award for outstanding work on behalf of Danish Children in 1985 and the International LEGO-Prize (“The Children’s Nobel Prize”) for an extraordinary contribution towards improvement in child welfare and well being in 1987. E-mail: jmerrick@internet-zahav.net. Website: www.nichd-israel.com
